# Systems biology of Ewing sarcoma: a network model of EWS-FLI1 effect on proliferation and apoptosis

**DOI:** 10.1093/nar/gkt678

**Published:** 2013-08-08

**Authors:** Gautier Stoll, Didier Surdez, Franck Tirode, Karine Laud, Emmanuel Barillot, Andrei Zinovyev, Olivier Delattre

**Affiliations:** ^1^Institut Curie, 26 rue d’Ulm, 75248 Paris cedex 05, France, ^2^INSERM U900, Bioinformatique, biostatistique et épidémiologie d’un système complexe*,* Paris, France, ^3^Mines ParisTech, Fontainebleau, France, ^4^INSERM U830, Unité de Génétique et Biologie des Cancers, Paris, France and ^5^Institut Curie, Unité de génétique somatique, Paris, France

## Abstract

Ewing sarcoma is the second most frequent pediatric bone tumor. In most of the patients, a chromosomal translocation leads to the expression of the EWS-FLI1 chimeric transcription factor that is the major oncogene in this pathology. Relative genetic simplicity of Ewing sarcoma makes it particularly attractive for studying cancer in a systemic manner. Silencing EWS-FLI1 induces cell cycle alteration and ultimately leads to apoptosis, but the exact molecular mechanisms underlying this phenotype are unclear. In this study, a network linking EWS-FLI1 to cell cycle and apoptosis phenotypes was constructed through an original method of network reconstruction. Transcriptome time-series after EWS-FLI1 silencing were used to identify core modulated genes by an original scoring method based on fitting expression profile dynamics curves. Literature data mining was then used to connect these modulated genes into a network. The validity of a subpart of this network was assessed by siRNA/RT-QPCR experiments on four additional Ewing cell lines and confirmed most of the links. Based on the network and the transcriptome data, CUL1 was identified as a new potential target of EWS-FLI1. Altogether, using an original methodology of data integration, we provide the first version of EWS-FLI1 network model of cell cycle and apoptosis regulation.

## INTRODUCTION

Ewing’s sarcoma is the second most frequent pediatric bone tumor with a peak of incidence between 4 and 25 years of age. In 85% of the patients, a causal translocation between EWS and FLI1 genes is found. This leads to the expression of EWS-FLI1 chimeric transcription factor (1). In most of the remaining patients, alternative translocations between EWS and another ETS- family member (ERG, FEV, ETV1, E1AF …) are detected. Ewing sarcoma presents a remarkable characteristic: its oncogenesis is generally accepted to be initiated by a single genetic event, i.e. one of the above mentioned translocations. Indeed, EWS-FLI1 alone has been shown to be able to transform NIH3T3 fibroblasts (2). Furthermore, expressing EWS-FLI1 in mouse mesenchymal stem/progenitor cell populations could recapitulate the disease *in vivo* (3,4). Moreover, knocking down EWS-FLI1 in Ewing sarcoma cell lines slows down proliferation and induces apoptosis *in vitro* (5) and *in vivo* (6). Finally, rescuing these two last phenotypes by re-expressing any other gene than EWS-FLI1 could not be accomplished so far. Therefore, Ewing sarcoma and EWS-FLI1 signaling can be seen as a primarily model for understanding cancer initiation and progression in a systemic manner. EWS-FLI1 has been reported to regulate cell cycle and apoptosis at various levels. For instance, EWS-FLI1 can modulate the cell cycle machinery by targeting directly p21/CDKN1A (7), Cyclin D (8,9) and Cyclin E (10) or indirectly through p57/KIP2 (11), TGFbeta- (12), IGF- (13,14) or MAPK signaling (15). The impact of EWS-FLI1 on apoptosis can be explained, for instance, by its direct effect on CASP3 (16) or indirectly through regulating members of TNF- (17), IGF- (13,14) and TGFbeta signaling (12).

Nonetheless, the global effect of EWS-FLI1 on cell cycle progression and apoptosis is still poorly understood. Indeed, classical approaches for elucidating the function of a gene usually look at upstream regulators and down-stream targets within a pathway, missing possible interplays with other pathways. Recent reports have started to address these issues by meta-analysis of genome-scale data to identify lists of the genes that are deregulated by EWS-FLI1 in Ewing’s sarcoma models (18) or linked to cell cycle regulation, proliferation, response to DNA damage and cell differentiation (19).

The above mentioned publications favor the point of view that EWS-FLI1 has a pleiotropic effect and should be considered in the context of a global gene regulation network. This justifies the usage of a systems biology approach (20): ultimately, such an approach produces an abstract model including deregulated genes and describing how these genes interact with each other (21). The signaling network regulated by EWS-FLI1 is specific to this disease and can be considered as the basis for its theoretical description. This description is possible because Ewing sarcoma is more genetically homogenous than other cancers where the choice of deregulated pathways is more difficult.

A valuable source of data for systems biology approaches is time-resolved response of perturbed experimental systems. These data allow constructing mathematical models describing time evolution of molecular networks and predicting their response to various perturbations (22). Time-series of transcriptome response to silencing/re-expressing of EWS-FLI1 were published in (23). However, these experiments did not allow to follow the transcriptome response for a time period longer than a few days, whereas significant transcriptome changes after EWS-FLI1 inhibition can be observed even after 1 week. Here, we took advantage of cell lines transformed with a tetracycline inducible shRNA system targeting EWS-FLI1 transcript (24) and collected long-term [inhibitory (17 days) and inhibitory (10 days)/re-expression(7 days)] transcriptional time series.

This article presents a network model dedicated to Ewing sarcoma: it describes EWS-FLI1 effect on proliferation and apoptosis. We decided to represent it through a ‘gene influence network’, as it is the only suitable representation for including incompletely characterized molecular interactions. This model was constructed in three steps: (i) Time-series data obtained in EWS-FLI1 modulated cell lines were analyzed. An original theoretical method was developed for selecting genes modulated by EWS-FLI1 and involved in cell-cycle regulation and apoptosis. (ii) An influence network was reconstructed from the literature connecting the above selected genes. (iii) Experimental validation of a part of the regulation network was performed in five Ewing cell lines. In addition, some additional transcriptional influences were identified by network reverse engineering using gene silencing data. These influences were compared with the literature-based network and confirmed its validity. This comparison also allowed to highlight EWS-FLI1 implication in the regulation of the ubiquitin proteasome system (through CUL1, SKP2 …) and to identify CUL1 as a novel direct target of EWS-FLI1.

The detailed description of the signaling involved in Ewing sarcoma oncogenesis should provide background for further theoretical search of *combinatorial* therapeutic strategies by predictive mathematical modeling, as it is done in other cancer studies (25).

## MATERIALS AND METHODS

### Transcriptome time series of shRNA-inducible Ewing cell lines

Tetracycline-inducible shRNA (directed against EWS-FLI1) clones shA673-1C and -2C (24) were used to perform a long-term inhibitory (t = 0–17 days) and inhibitory (t = 0–10 days)/rescue (t = 10–17 days) time series experiments. EWS-FLI1 invalidation was achieved by adding 1 µg/ml of doxycycline in the cell culture media. Cells were split twice a week. For the inhibitory time series, RNAs were collected at day 0, 1, 2, 3, 6, 9, 11, 13, 15, 17 after addition of doxycycline to the media. For the rescue time series, doxycycline was omitted from the media after 10 days and RNAs were collected at day 13, 15 and 17. Total RNAs were isolated using the Trizol Reagent (Invitrogen) at the different time points. EWS-FLI1 silencing and re-expression was validated by real-time quantitative reverse transcription-PCR as previously described by Tirode *et al.* (24). Gene expression profiles of the time series experiments were assessed by microarray profiling using Affymetrix HG-U133plus2 arrays (Affymetrix, Inc., Santa Clara, CA). Experimental procedures for cRNA target synthesis and GeneChip microarray were done according to the standard protocols described by Affymetrix Company.

### Fitting non-linear response models to the time series

Points of time series were fitted by two types of curves:

(i) Hyperbolic tangent: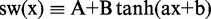 (a ‘switch’ with four parameters)
(ii) Generalized Gaussian: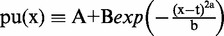 (a ‘pulse’ with five parameters)

For the temporal response of each probeset in each clone, the hyperbolic tangent was fitted in the case of simple inhibition of EWS-FLI1 and the generalized Gaussian in the case of inhibition/re-expression of EWS-FLI1. The score for each fit is the ratio between an *amplitude α* and a *mean-squared error δ* multiplied by a *transition time penalization factor* τ:

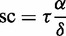



The *mean-squared error δ* is the square root of the sum of squared differences between the curve and data points. The *amplitude α* is the difference between the high and low expression levels. These levels are defined as follows:

- For the hyperbolic tangent (‘switch’), the inflexion point of the curve define naturally a transition time separating the time points in a high level and a low level window. The two levels are simply the averages of data points on the two windows defined above.
- For the generalized Gaussian (‘pulse’), the two inflection points of the curve define three time windows. We merge the first and the last one and obtain two windows. Levels are computed by averaging as in (i).

The *transition time penalization factor* τ is given by the following formulas:

(i) For the hyperbolic tangent (‘switch’),

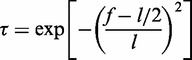

where *f* is the position of the inflection point and *l* the length of the time window.
(ii) For the generalized Gaussian (‘pulse’),

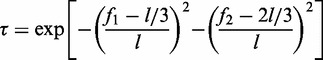

where *f*_1_, *f*_2_ are the two inflection points and *l* is the length of the time window.

If one inflection point is outside the experimental time window, it is artificially shifted inside in order to be between the first and the second time points or the last and one before last time points. If there are no time points between the two inflexion points of the generalized Gaussian, the inflection points are artificially shifted away to the closest time points. If the extremum of the generalized Gaussian (parameter *t*) is outside the experimental time window, the score is simply set to 0. See Figure 3C for illustrations of these fitness scores.

As a result of this quantification procedure, the response of every gene (probeset) on the Affymetrix chip can be characterized by few parameters having clear interpretation: switching time, switching speed, re-expression time, re-expression speed, and the scores for switch-like and pulse-like model curves (Supplementary Table S1 and Figure 3C for examples). All these parameters can be used for functional characterization of a group of genes. The curve fitting was performed in MATLAB, using MATLAB Curve Fitting toolbox.

### Protocol for selecting genes for network reconstruction

The selection of genes and pathways were based on three steps:

- Selecting genes according to the fitness score in transcriptome time series experiments: we selected 3416 genes that have fitness score higher than a given threshold, in both inhibition and inhibition/re-expression experiments and in at least one clone for at least one probeset (3033 probesets only in clone shA673-1C, 1003 only in clone shA673-2C, 867 probesets in both clones, 4903 probesets in total). The thresholds were 10% lower than the minimum score value of a sample of probesets, selected by visual inspection of their time series (histograms of scores and thresholds are given in Supplementary Figure S2).
- Reducing the list produced in (i) using GO (26) and BROAD/MSigDB (27) annotations: we reduce the list to the genes having associated GO terms ‘cell cycle’ and ‘apoptosis’. We also consider the genes selected in (i) that belong to the following BROAD terms: ‘cell cycle arrest’, ‘cell cycle checkpoint’, ‘cell cycle pathway’, ‘apoptosis’ (see Supplementary Table S1). A list of 407 genes was obtained using this filtering approach (a heat map of these gene expressions in provided in Supplementary Figure S7). These genes are clearly separated in two groups: those activated on DOX treatment, those inhibited on DOX treatment.
- Consider only genes/pathways whose effect can be assembled in an influence network: among the list of genes of (ii), we consider only a subpart, whose effects on proliferation or apoptosis has been studied enough in order to be assembled in a connected network (37 genes).

In parallel, we selected only those gene sets that have been shown to be significantly enriched in GSEA analysis (with nominal *P*-value < 1%). Furthermore, we consider only those pathways that have been shown to be involved in controlling directly cell proliferation and apoptosis. These selected pathways are highlighted in red in Supplementary Tables S2–S5. Final results of both selections methods are summarized in Table 1.

**Table 1.** Selected pathways

Pathways	Criteria	Method of selection
Tumor Necrosis Factor	Some of members of TNF families, including TNF receptors are negatively influenced by EWS-FLI1 in A673 cell line. In addition, it has been shown in that TNF pathway is regulated by EWS-FLI1 (17).	Genes selection
Transforming growth factor beta	TGFB2 and some of TGFB receptors are negatively induced by EWS-FLI1 in A673 cell line. SMAD target gene sets are enriched according to the GSEA analysis. TGFBR2 has been identified as a direct target of EWS-FLI1 (12).	Genes selection
		GSEA
MAP kinase	ERK and JNK members are negatively induced by EWS-FLI1. In addition, MAPK kinases have connections to other pathways (TNF, Myc) and are known to be a major factor affecting the cell fate decision between apoptosis and proliferation.	Genes selection
IGF	Although mRNA of IGF1 and IGF2 are not clearly influenced by EWS-FLI1, IGFBP3 is negatively induced by EWS-FLI1 in A673 cell lines and have been identified as a direct target. In addition, IGFBP3 is known to be a direct target of EWS-FLI1 (14).	Genes selection
NfkB	One of the available NFkB pathway signatures is enriched in GSEA analysis. Moreover, NFkB pathway is known to be induced by TNF. In addition, it has been shown that NFkB pathway is regulated by EWS-FLI1 (17).	GSEA
c-Myc	MYCBP (‘c-myc bind protein’, a c-myc activator) is positively induced by EWS-FLI1 in A673 cell line. In addition, several Myc-related gene sets are enriched in GSEA analysis. Myc has also been shown to be regulated by EWS-FLI1 (11).	Genes selection
		GSEA
Apoptosis	Many genes are influenced by EWS-FLI1, like CASP3 and CYCS. In addition, several gene sets that are related to apoptosis are enriched in GSEA analysis.	Genes selection
		GSEA
Cell-cycle	Many of the genes involved in cell-cycle machinery (like cyclins, cyclin inhbitors, degradation complexes, key transcription factors) are influenced by EWS-FLI1. In addition, targets of E2Fs and cell-cycle regulation gene sets are enriched in the GSEA analysis. In addition, these genes have been identified as being directly regulated by EWS-FLI1, like p21/CDKN1A (7), Cyclin D (8,9) and Cyclin E (10).	Genes selection
		GSEA
PDGF	Enriched in GSEA analysis	GSEA

Arguments explaining the reason for including the pathway in network reconstruction are given together with references to publications identifying those pathways.

### Network curation framework: construction of the fact sheet

This step consists in the construction of a textual description (‘interaction fact-sheet’) of pseudo-reactions describing the influences between biological ‘entities’: genes, proteins, proteins families, modified proteins (e.g., by phosphorylation) or complexes. An extract of the fact-sheet is given in Table 2. The whole fact sheet is available in Supplementary Tables S7 and S8.

**Table 2.** A subset of the fact sheet used to construct the network

ReviewRef	ExperimentRef	Link	ChemType	Delay	Confidence	Tissue	Comments
	PMID:10074428	TRAF2 → (NFKB.)	Influence	12 h	0.7	TRAF2 mutant Embryonic Kidney 293 cells	Activation of NFKB by TNFS18 was observed 24 h later
	PMID:10074428	MAP3K14 → (NFKB.)	Influence	12 h	0.7	MAP3K14 mutant Embryonic kidney 293 cells	Activation of NFKB by TNFS18 was observed 24 h later*,* (other name for MAP3K14: NIK)
PMID:12887914		TNFRSF1A → (TNFRSF1A:RPAIN)	Binding		0.8		Complex I formation, (other name for TNFRSF1A: TNF-R1), (other name for RPAIN: RIP)
PMID:12887914		TNFRSF1A → (TNFRSF1A:TRAF2)	Binding		0.8		Complex I formation, (other name for TNFRSF1A: TNF-R1)
PMID:12887914		(TNFRSF1A:RPAIN) → (NFKB.)	Post-transcriptional influence		0.7		(other name for TNFRSF1A: TNF-R1), (other name for RPAIN: RIP)
PMID:12887914		(TNFRSF1A:TRAF2) → (NFKB.)	Post-transcriptional influence		0.7		(other name for TNFRSF1A: TNF-R1)
PMID:16502253		TNFRSF1A → CTSB	Release		0.6		TNFR permeablized the lysosome membrane, release CTSB, true for other cathepsin, (other name for TNFRSF1A: TNF-R1)
PMID:16502253		CTSB → BID	Cleavage		0.8	In vitro	Bid induce apoptosis through mitochondria and CASP9
PMID:16502253		(NFKB.)-|CTSB	Post-transcriptional influence		0.7		Through SPIN2A, figure

PMID:16502253		CASP8 → CTSB	Release		0.6	Hepatocyte	Through lysosome release
PMID:16502253		CTSB → [apoptosis]	Chromatin condensation		0.7	Cell-free systems	
PMID:16502253		CTSB → BAX	Influence		0.4	Mutant mice	Hypothetical connection could explain BID free apoptosis induced by CTSB

Titles of the column are given in the first line. The ‘confidence’ is a number between 0 and 1 indicating subjective reliability of the regulatory connection. Genes are named accordingly to HUGO, names of the complexes are enclosed into parenthesis, with component names separated by colon; names of the families of genes are enclosed into parenthesis, with family members separated by comma or defined by a wildcard: for example, (NFKB.) notifies the family consisting of NFKB1, NFKB2 etc.

### Network curation framework: implementing the fact sheet in Cytoscape

To construct the influence network enriched with the genes responsive to EWS-FLI1 inhibition/re-expression from the fact sheet, we developed a software, integrated into the BiNoM Cytoscape plugin (28). BiNoM is capable of processing the fact sheet described above in a self-consistent way, providing an interface to the user who decides on what level of abstraction to represent the entities (in the form of a family or an individual family members). At the second step of the pre-processing, the implicit reactions needed for consistent representation are added to the network, also under the user control. The actual facts sheet used for the Ewing’s cancer network together with pre-processing protocol is provided in the web page of Supplementary Material (‘Processing the fact sheet’). This web page includes the final network provided as a Cytoscape session file and a BioPAX file with all annotations from the fact sheet.

### siRNA, RT-QPCR, Western Blots and ChIP procedures

Experimental procedures and references for siRNA, RT-QPCR, ChIP and Western blots as well as primers and antibodies used for these experiments are detailed in Supplementary Table S9.

### Network reverse engineering from siRNA silencing data

In the first step, influences are inferred from siRNA/RT-QPCR experiments. For that, a linear mixed model has been implemented in R (lme package), to determine linear dependence between presence of siRNA (two discrete levels) and gene expression, considering the fluctuations due to the difference between the clones and RT-QPCR measurement noise. All siRNAs significantly silenced their targets (*P*-value smaller than 1.5 × 10^−^^7^). Therefore, this *P*-value was chosen as a threshold for identifying influences. All connections extracted from the literature (Figure 6A) were confirmed by this method.

In the second step, the inferred influences were separated into *necessary* and *non-necessary* connections, using the sub-network from Figure 6B. In that context, *non-necessary* connections are links that can be explained by a signed path in the sub-network containing at least one intermediate node. Any other connection is said to be *necessary*.

In the third step, we applied again the concept of necessary connections, using the whole influence network shown in Figure 4A as *network model* (see the definition of necessary connection in supplementary Figure S3). Using this network, we checked the solid arrows in Figure 6B for their necessity (the results are listed in Table 4). Only one influence, EP300 -| E2F2, remained necessary after this test. This is not surprising given the fact that the network from Figure 4A is larger than a reconstructed subnetwork from Figure 6B; hence, it contains more paths that can indirectly explain the inferred influences.

## RESULTS

**Figure 1.**
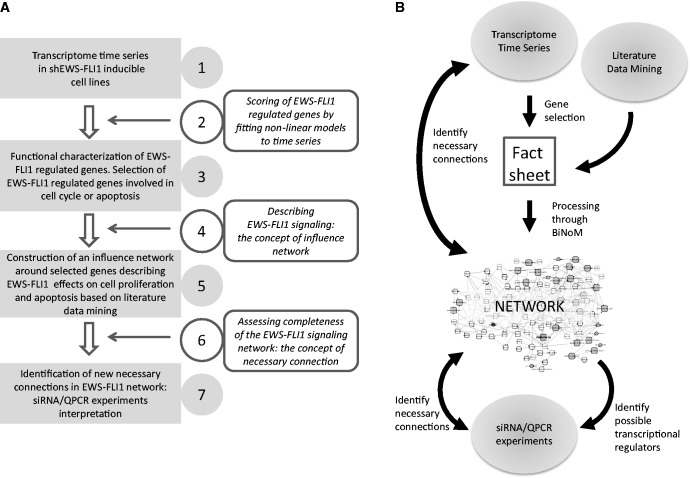
(**A**) Flow chart of the article. Gray rectangles are key steps of our analysis. Methods and concepts are described in rounded rectangles. (1) Transcriptome time-series data were obtained from shA673-1C and -2C clones after silencing or silencing and re-expressing EWS-FLI1. (2) An original method based on nonlinear curve fitting was used to perform the analysis of transcriptome time series. (3) EWS-FLI1-modulated genes were selected; this list was restricted to the genes affecting proliferation and apoptosis. (4) A network representation of EWS-FLI1 signaling was chosen; it consists of influences (positive or negative) between genes, proteins and complexes. (5) EWS-FLI1 signaling network model was reconstructed from the above selected genes connected by the influences known from literature. (6) The notion of necessary connection was introduced; it allows to refine a network model when, for instance, additional experimental data are provided. (7) Silencing experiments were performed on several EWS-FLI1-regulated genes; new necessary connections were identified and added to EWS-FLI1 signaling network. (**B**) Causal relations between data and the influence network.

The starting point of this study was the statement that EWS-FLI1 is the central and driving force of tumorigenesis in Ewing sarcoma. To better understand long-term downstream effects of EWS-FLI1, shA673-1C and shA673-2C tetracycline-inducible cell lines in which EWS-FLI1 can be silenced and re-expressed were used (24). The flow chart of our approach is illustrated in Figure 1A, and the causal relations between data and the influence network is represented in Figure 1B. The principle was to combine transcriptome time series obtained *in vitro* with literature data mining to construct a first version of the influence network dedicated to Ewing sarcoma focused on regulation of apoptosis and proliferation by EWS-FLI1.

### Transcriptome time series in shEWS-FLI1 inducible cell lines

**Figure 2.**
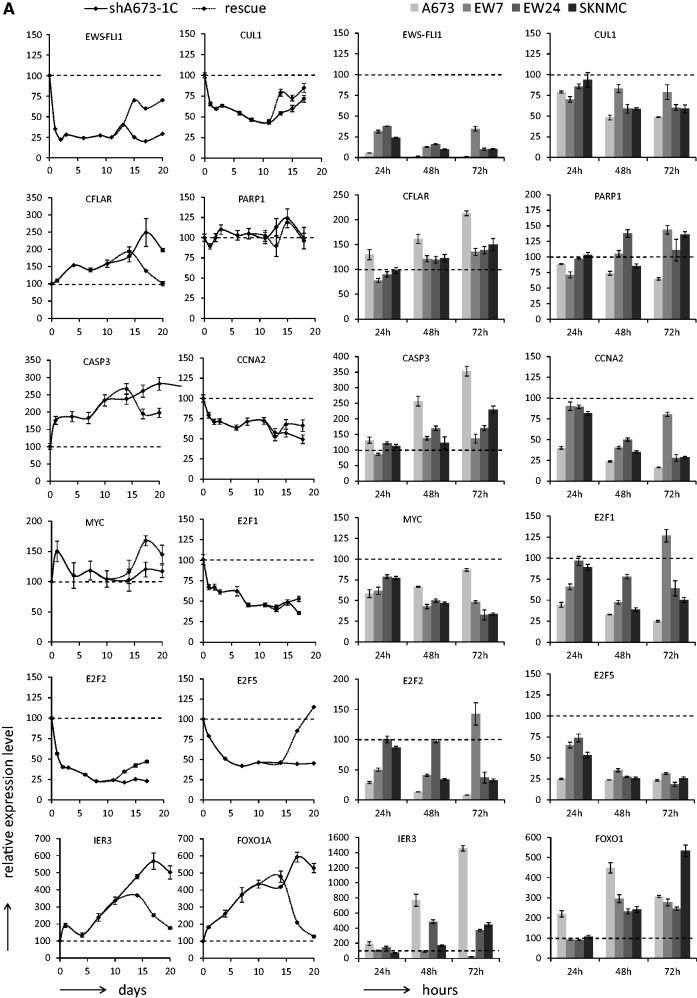

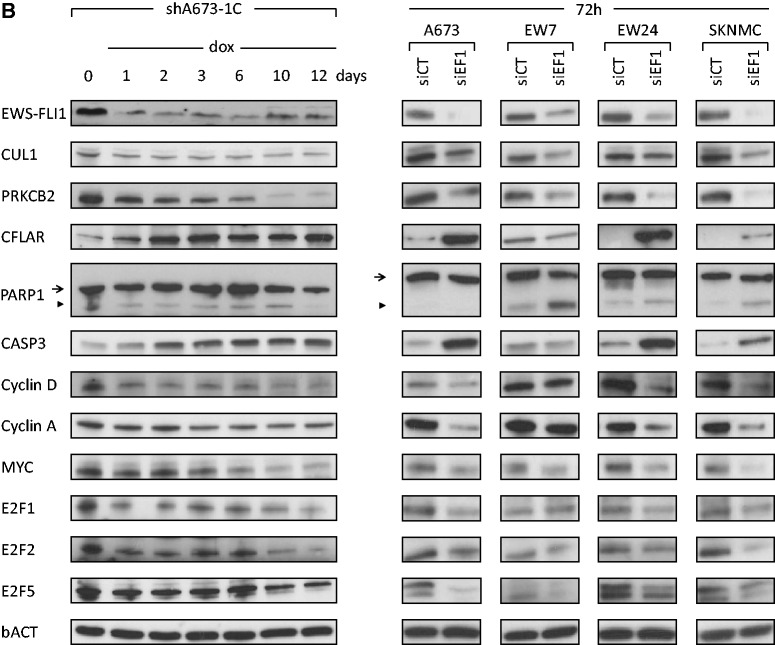
(**A**) RT-QPCR for a panel of EWS-FLI1-modulated genes along time series experiments in shA673-1C cells on DOX addition/removal (solid: inhibition, dashed grey: rescue) and in four Ewing cell lines (A673, EW7, EW24 and SKNMC) on transfection with nontargeting siRNA (siCT) or EWS-FLI1-targeting siRNA (siEF1) after 24, 48 or 72 h. Relative expression level (%) for each gene to the starting point shA673-1C condition or to siCT conditions are displayed on the y axis. Data are presented as mean values and the standard deviations. (**B**) Western blot for a panel of EWS-FLI1-modulated genes along a time series experiment in shA673-1C cells on DOX addition and in four Ewing cell lines (A673, EW7, EW24 and SKNMC) on transfection with nontargeting siRNA (siCT) or EWS-FLI1 targeting siRNA (siEF1) after 72 h. For PARP western blot, full length protein is indicated by the arrow and cleaved PARP by the arrowhead. Beta-actin was used as loading control.

A time-series experiment was performed with both shA673-1C and shA673-2C clones by adding doxycycline (DOX) to the media from day 1 to 17. In addition, a rescue time-series experiment was also performed from day 10 to 17 by withdrawing DOX from the culture medium. Transcriptomic profiles were generated from these experiments. Stable and similar inhibition of EWS-FLI1 was observed in both clones on addition of DOX (Figure 2 and Supplementary Figure S1).

### Scoring EWS-FLI1 regulated genes by fitting non-linear models to time series

**Figure 3.**
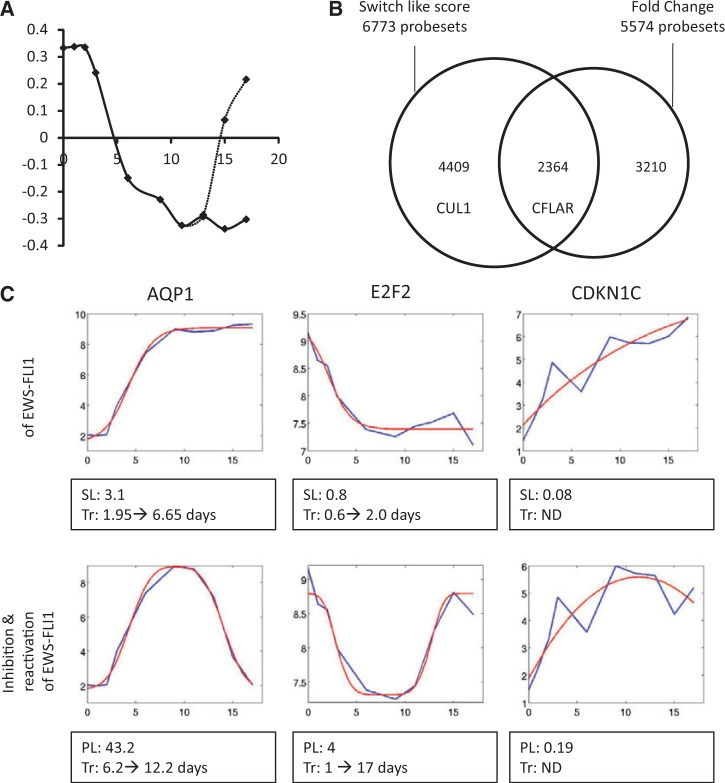
(**A**) Time series corresponding to the first principal modes of gene expression variation in EWS-FLI1 inhibition (solid line) and re-expression experiments (dashed line). (**B**) Comparison of two methods for selecting modulated genes, one based on switch like (SL) score, the other one based on fold change (FC). For both methods, top 4000 probesets for each clone (shA673-1C and -2C) were selected (ranked by their SL score or by FC between the first and the last time points). The Venn diagram compares these top scored probesets. The intersection of both methods is partial for two reasons: (i) the SL score can be large for a time series tightly following the assumed model of response, even if having a moderate variance, (ii) FC method is not considering intermediate time points. Both CUL1 and CFLAR exhibit temporal expression responses that have a good fit to the proposed switch-like response model. However, only some CFLAR probesets are characterized by significant fold change values. (**C**) Examples of curve fitting to the time series in microarray experiments. AQP1, E2F2 and CDKN1C expression profiles are shown. Blue curves represent microarray experimental values; red curves correspond to fitted functions. Switch-like scores (SL), pulse-like scores (PL) and transitions parameters (Tr) are listed under each plot. SL and PL scales are not comparable as the fitting procedures are different. It can be noticed that both scores for E2F2 are smaller than those for AQP1 for two reasons: the amplitude of expression variation is smaller for E2F2 and the transition happen at a time point closer to the limits of the time window. The scores for CDKN1C are clearly lower, because the expression level is less smooth. In that case, transition parameters cannot be identified, because the inflections points of the fitted curves are outside of the time window.

At first, we performed simple PCA analysis of time-series, aiming at obtaining the dominant modes of gene expression variation similarly to the work of Alter *et al.* (29). 942 microarray probesets with (i) highly correlated expression profile in both clones (Pearson correlation coefficient >0.85) and (ii) a significant variation in both clones (geometrical mean variation bigger than the 95th percentile) were selected. These last probesets were then used to perform the PCA. The time series corresponding to the first principal component (explaining 57% of data variance) for the inhibition and re-expression experiments are shown in Figure 3A. This indicates that the switch-like (single transition) and pulse-like (double transition) modes of gene expression variation are predominant in such EWS-FLI1 inhibition and re-expression experiments. Therefore, an original method was developed to automatically and systematically characterize gene expression profiles on EWS-FLI1 inhibition/re-expression. Two time series models were considered: (i) one curve describing the *switch-like* (SL, single transition) profile, applied to EWS-FLI1 inhibition (DOX+); (ii) one curve describing *pulse-like* (PL, double transition) profile, applied to EWS-FLI1 inhibition/re-expression (DOX+, DOX−). A fitness score was computed for time series of each probeset which defines the accuracy of the fit (the ratio between estimated amplitude and the mean-squared error of the fit). Four scores were generated for each probeset (switch-like score (SL) and a pulse-like score (PL) for both shA673-1C and -2C clones). Fitness score distributions are shown in Supplementary Figure S2. A threshold for the switch-like score (tsh^SL ^= 0.024) and the pulse-like score (tsh^PL ^= 0.94) were set using careful manual inspection of many individual profiles (see Materials and Methods and Supplementary Figure S2). By definition, a gene was selected for further analysis if both SL and PL scores were higher than their respective thresholds in at least one clone and for at least one probeset. Global EWS-FLI1 transcriptional response is slightly different between the two clones: fitness scores are higher in clone shA673-1C. The interest of this procedure is that (i) high fitness scores can correspond to high amplitude of expression but also to small amplitude response that tightly fit the model curve; this avoids a bias in selecting highly expressed genes; (ii) parameters describing transition time and speed are not predefined, they are identified from the data (Figure 3C, Supplementary Table S1 and Supplementary Figure S2); they are not based on a given dynamical model (like ODE). Our method is clearly different from the standard fold change-based gene selection approach, as illustrated in Figure 3B. Therefore, genes with high fitness score were hypothesized to be potentially modulated by EWS-FLI1. It is to be noted that the fitness scores (SL = 0.667 and PL = 8.72) of the first principal components (Figure 3A) are substantially larger than the respective threshold values (see above).

### Functional characterization of EWS-FLI1 regulated genes

The characterization of EWS-FLI1 regulated genes was based on two approaches.

In the first method, GSEA method, using MSigDB (27), was applied separately to the four fitness scores computed for all probesets. Enriched pathways resulting from these four GSEA analyses are listed in Supplementary Tables S2–S5.

In the second method, DAVID tool (30,31) was applied to the lists of modulated genes. 3416 genes (4903 probesets) were selected as potentially modulated by EWS-FLI1 (1426 inhibited and 1990 induced, listed in Supplementary Table S1). DAVID functional annotation tool was applied to the list of modulated genes to produce a list of enriched pathways based on GO, KEGG and REACTOME annotations (Supplementary Table S6).

Both functional characterization methods result in identification of multiple pathways potentially implicated in response to EWS-FLI1 inactivation. As expected, such categories as cell cycle regulation, RNA processing and cell death clearly showed up. We decided to focus on proliferation and apoptosis because, in addition to our bioinformatics analysis, previous reports also clearly support this decision. Indeed, EWS-FLI1 knock-down inhibits proliferation in our cellular model and in other Ewing cell lines (5) and can also drive cells to apoptosis (14,32).

### Describing EWS-FLI1 signaling: the concept of influence network

An important objective of this study is to understand how the genes and pathways modulated by EWS-FLI1 interact with each other. The above described analysis only allowed selecting genes whose temporal expression profiles can be fit to a simple switch/pulse-like function. To reconstruct a mechanistic picture of causal relations, EWS-FLI1 must be integrated in a complex regulatory network, where the modulated genes are connected together through interactions with other intermediate genes that are not necessarily modulated by EWS-FLI1. Such a gene regulation network represents a first step toward modeling, and therefore understanding the EWS-FLI1 signaling.

Ideally, an exhaustive representation including biochemical processes and phenotypic outcomes for all genes/pathways should be integrated in this network. For instance, ‘comprehensive’ network maps of EGFR and RB signaling (33,34) have been constructed, including more than a hundred proteins and genes. However, applying similar approach to describing EWS-FLI1 signaling is not suitable. Firstly, the number of genes/pathways involved here is large (see GSEA results, Supplementary Tables S2–S5), while above mentioned RB and EGFR signaling network maps describe only one pathway. The resulting ‘comprehensive’ network would be difficult to manipulate. Secondly, many of the selected genes/pathways are poorly described and therefore difficult to connect in a ‘comprehensive’ network. Therefore, we decided to construct an *influence network* (35). By definition, edges in the influence network can only represent positive or negative induction (Supplementary Figure S3). In the context of our study, nodes can represent mRNAs, proteins or even complexes. Hence, this allows to integrate both well characterized as well as poorly described biological interactions.

### Construction of an influence network describing EWS-FLI1 effects on cell proliferation and apoptosis based on literature data mining

The *influence network* was reconstructed around the regulation of proliferation and apoptosis using EWS-FLI1-modulated genes. The list of 3416 modulated genes (selected above) was shrunk to the genes known to have a role in regulation of proliferation or apoptosis, according to GO (26) and BROAD/MSigDB databases (27). This list was further reduced to 37 genes whose mechanisms of cell cycle and apoptosis regulation are clearly documented in the literature (top probesets of Supplementary Table S1, labeled by ‘Net reconst’). Enriched pathways affecting proliferation/apoptosis and selected by GSEA were also included (highlighted in red in supplementary Tables S2–S5). This pathway (or set of genes) selection procedure is detailed in Materials and Methods, in ‘Protocol of selecting genes for network reconstruction’. Table 1 lists the eight pathways used for network reconstruction together with the criterion used for their selection (EWS-FLI1 modulated genes selected by curve fitting method and/or by GSEA).

**Figure 4.**
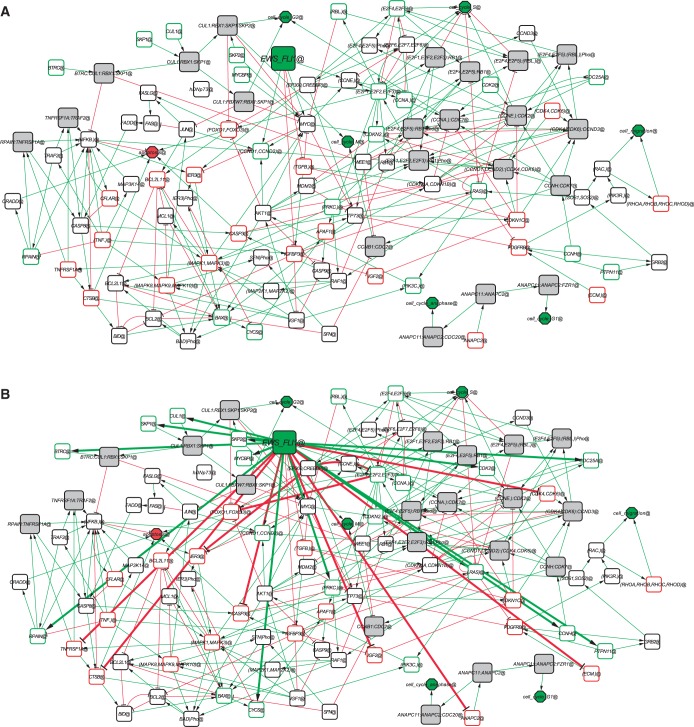
(**A**) Annotated network of EWS-FLI1 effects on proliferation and apoptosis, derived from literature-based fact sheet. White nodes represent genes or proteins; gray nodes represent protein complexes. EWS-FLI1 (green square) and cell cycle phases/apoptosis (octagons) represent the starting point and the outcome phenotypes of the network. Green and red arrows symbolize respectively positive and negative influence. Nodes with green frame are induced by EWS-FLI1 according to time series expression profile and nodes with red frame are repressed. The network structure shows intensive crosstalk between the pathways used for its construction, up to the point that the individual pathways cannot be easily distinguished. (**B**) Refined network including new links inferred from experimental data (thick arrows) from transcriptome time series and siRNA/RT-QPCR.

The network construction was then achieved in two steps. Firstly, an *interaction fact sheet* was generated*:* this sheet is a description of annotated influences extracted from the literature (around 400 influences); a sub-part of it is given in Table 2 (the full table is given in Supplementary Tables S7 and S8), illustrating the formalism for interpreting a publication in terms of influence(s) between genes/proteins. Secondly, a graphical representation of the network extracted from the fact sheet was produced. The later step allows to handle gene families (i.e. E2Fs, IGFs) and to add implicit connections (for instance, CDK4 positively influences the (CDK4:CCND) complex formation) (see *Network curation framework* in Materials and Methods and Protocol 1 in the web page of supplementary material). The fact sheet was confronted to the TRANSPATH database (36), and missing links were manually curated and included. The advantage of this procedure is its flexibility: it is easy to update the fact sheet with new publications and to produce a new version of the network. The resulting influence network is shown in Figure 4A and is accessible as a Cytoscape (37) session file, available at http://bioinfo-out.curie.fr/projects/suppmaterials/suppmat_ewing_network_paper/Supp_material/Network/Suppl_File_1_Net_1_CytoscapeSession.cys. This network contains 110 nodes and 292 arrows (213 activations and 79 inhibitions). Annotations from the fact-sheet can be read using the BiNoM plugin (BioPAX (38) annotation file is available at http://bioinfo-out.curie.fr/projects/suppmaterials/suppmat_ewing_network_paper/Supp_material/Network/Suppl_File_2_Net_2_BIOPAX_Annotation.owl).

This network can be seen as an organized and interpreted literature mining (43 publications, mainly reviews, listed in the fact sheet, Supplementary Table S8). It includes schematic description of the crosstalk between the following signaling pathways: apoptosis signaling (through the CASP3 and cytochrome C), TNF, TGFβ, MAPK, IGF, NFκB, c-Myc, RB/E2F and other actors of the cell-cycle regulation. Many of the pathways that were identified in this influence network have been previously described or discussed in the context of Ewing sarcoma. During reconstruction of the network, 9 genes regulated by EWS-FLI1 were added to the 37 genes identified from the selection procedure (Supplementary Table S1).

### Experimental validation of EWS-FLI1 modulated genes

To assure biological significance of this Ewing sarcoma network, a substantial number of EWS-FLI1 modulated genes were assessed by RT-QPCR (Figure 2A) and western blotting of the corresponding proteins (Figure 2B) using DOX time series experiments in the shA673-1C clone. To further validate these results, siRNA time series experiments (24, 48 and 72 h) with siEWS-FLI1 (siEF1) and control siRNA (siCT) were performed in four additional Ewing cell lines (A673, EW7, EW24 and SKNMC). As expected, cyclin D (8,9) and protein kinase C beta (39) proteins (two direct EWS-FLI1 targets genes) were down-regulated in these cell lines upon EWS-FLI1 silencing (Figure 2B). MYC was previously shown to be induced by EWS-FLI1 most probably through indirect mechanisms (11). This was confirmed here at the protein level in all tested cells (Figure 2B). Down-regulation of MYC mRNA was also observed upon siRNA treatment in all cell lines. The MYC variation was less obvious in the DOX-treated shA673-1C clone probably due to the milder inhibition of EWS-FLI1 by inducible shRNA (Figure 2A) than by siRNA (supplementary Table S10). In addition to the previously published induction of Cyclin D (8,9) and Cyclin E (10) by EWS-FLI1, we report here the down-regulation of Cyclin A upon EWS-FLI1 silencing (Figure 2). Among other well described cell cycle regulators, E2F1, E2F2 and E2F5 were also consistently down-regulated after silencing of EWS-FLI1. Altogether, these results emphasize the strong transcriptional effect of EWS-FLI1 on various cell cycle regulators. Apoptosis was also investigated upon EWS-FLI1 inhibition. A clear up-regulation of procaspase3 (mRNA and protein) was observed in all cells (except for EW7 cells). To monitor late stage of apoptosis, induction of cleaved PARP was assessed upon EWS-FLI1 inhibition. No induction of apoptosis could be observed along the time series experiment in the shA673-1C (Figure 2B*, arrowhead band)*. This was probably due to the relatively high residual expression of EWS-FLI1 (20–30% of original levels, Figure 2). However, in the transient siRNA experiments where EWS-FLI1 was more efficiently knocked-down, apoptosis was monitored by induction of cleaved PARP in EW7, EW24 and SKNMC but not in A673 (Figure 2). It is to notice that full length PARP1 protein was not modulated upon silencing of EWS-FLI1 (Figure 2B, *arrow band)*. Interestingly, after EWS-FLI1 silencing, the potent anti-apoptotic CFLAR protein was strongly up-regulated in A673 but not in EW7 cells (Figure 2B). Phenotypically, this was associated with a strong induction of apoptosis and dramatic reduction of EW7 cell number but only mild effect on A673 proliferation (Supplementary Figure S4).

### Assessing completeness of the EWS-FLI1 signaling network: the concept of necessary connection

In the previous paragraphs, experimental data were used to select genes and to validate their biological implications. However, the connections in the network were extracted from the literature that is not always dedicated to Ewing sarcoma. Genes like IGFBP3, MYC and Cyclin D are linked to EWS-FLI1 because these influences have been reported (8,9,11,14). However, several genes (E2F5, SKP2 …) are modulated by EWS-FLI1 but are not directly linked to EWS-FLI1 (Figure 4A). Therefore, the network needs to be refined to match the context of Ewing sarcoma. To answer this question, we introduced the concept of *necessary connection* between genes. By definition, a necessary connection is such a regulatory connection between two molecular entities, which can be inferred from ‘the data’ but cannot be predicted from ‘already existing network model’. From its definition, a necessary connection always depends on (i) dataset, and (ii) already existing model. We provide in Supplementary Figure S3 several examples of necessary connections (always applying the same definition), for various practical situations. For instance, the connection ‘EWS-FLI1 → CUL1’ is necessary in our context (data and network) because (i) CUL1 is induced by EWS-FLI1 according to the transcriptome time series, (ii) no connection to CUL1 explains the transcriptional regulation of this gene in the network of Figure 4A. We decided to formalize this notion of necessary connection to handle the network model that can be incomplete (missing nodes and connections representing indirect effects). Subsequently, this definition was applied to all modulated genes in the network: the resulting necessary connections are listed in Table 3.

**Table 3.** Necessary connections between EWS-FLI-1 and the network genes

Node	Genes	Link
ANAPC2	ANAPC2	EWS-FLI1 -| ANAPC2
BTRC	BTRC	EWS-FLI1 → BTRC
CASP3	CASP3	EWS-FLI1 -| CASP3
CCNH	CCNH	EWS-FLI1 → CCNH
CDC25A	CDC25A	EWS-FLI1 → CDC25A
CDK2	CDK2	EWS-FLI1 → CDK2
(CDK4,CDK6)	CDK4,CDK6	EWS-FLI1 -| (CDK4,CDK6)
CTSB	CTSB	EWS-FLI1 -| CTSB
CUL1	CUL1	EWS-FLI1 → CUL1
CYCS	CYCS	EWS-FLI1 → CYCS
(E2F1,E2F2,E2F3)	E2F2	EWS-FLI1 → (E2F1,E2F2,E2F3)
(ECM.)	ECM1	EWS-FLI1 -| (ECM.)
IGF2	IGF2R	EWS-FLI1 -| IGF2
(.RAS)	KRAS	EWS-FLI1 → (.RAS)
MYCBP	MYCBP	EWS-FLI1 → MYCBP
(PRKC.)	PRKCB	EWS-FLI1 → (PRKC.)
PTPN11	PTPN11	EWS-FLI1 → PTPN11
RPAIN	RPAIN	EWS-FLI1 → RPAIN
SKP1	SKP1	EWS-FLI1 → SKP1
SKP2	SKP2	EWS-FLI1 → SKP2
TNFRSF1A	TNFRSF1A	EWS-FLI1 -| TNFRSF1A

The given data are the transcriptome time series, the given network is the reconstructed network based on literature. These connections target EWS-FLI1-regulated genes (identified by transcriptome time series) that have no identified transcriptional regulators.

Among these, several necessary connections between ubiquitin proteasome system members (CUL1, SKP1, SKP2, ANAPC2) and EWS-FLI1 were identified, potentially indicating an interesting link between this oncogene and the protein turnover regulation in the context of Ewing sarcoma. Necessary connections between EWS-FLI1 and two attractive candidates for their obvious implication in oncogenic process, the GTPase (KRAS) and the protein kinase C (PRKCB) were also identified using this approach. Finally, a set of necessary connections from EWS-FLI1 to cell cycle regulators (CDK2, CDK4, CDK6) or apoptosis members (CASP3, CTSB) were highlighted. To verify if these necessary connections were potentially direct, previously published FLI1 ChIPseq experiments performed on Ewing cell lines were examined for the presence of peaks around these target genes (40–42). A significant ChIPseq hit corresponding to a potential ETS binding site was found within the CUL1 gene. Interestingly, CASP3, here identified as EWS-FLI1 necessary connection, was identified as a direct target of EWS-FLI1 (16). However, no significant ChIPseq hit could be identified in the CASP3 promoter. This may be attributed to the relatively low coverage of the ChIPseq data used in this study. Eleven of the genes having a necessary connection to EWS-FLI1, with low CHIPseq read density within their promoter regions, were selected and assessed by ChIP (Supplementary Figure S5A and Supplementary Table S9). In agreement with published ChIPseq data, only CUL1 was identified as a direct target of EWS-FLI1 (see Supplementary Figure S5B). As indicated by the transcriptome time-series experiments, RT-QPCR and Western blot experiments confirmed that EWS-FLI1 induces CUL1. Indeed, the level of CUL1 is reduced to ∼50% on addition of DOX in the shA673-1C clone at both mRNA (Figure 2A) and protein level (Figure 2B). These results were confirmed in four additional cell lines using siRNA time series experiments (24, 48 and 72 h) and are shown in Figure 2.

### Identification of new necessary connections in EWS-FLI1 network: siRNA/RT-QPCR experiments interpretation

The *necessary connections* listed in Table 3 make the network compliant with the transcriptome time series results. To further understand EWS-FLI1 transcriptional activity, new experiments were required. We focused on three EWS-FLI1 regulated genes: FOXO1A, IER3 and CFLAR. These genes were selected because they participate to the regulation of the cell cycle and apoptosis machinery although their transcriptional regulation is not yet fully elucidated. FOXO1A regulates cell cycle mainly through P27(kip1) (43) and is connected to apoptosis by regulation of TRAIL (44), FASL and BIM (45). IER3 is a modulator of apoptosis through TNF- or FAS-signaling (46) and MAPK/ERK pathway (47). CFLAR is a potent anti-apoptotic protein that share high structural homology with procaspase-8 but that lack caspase enzymatic activity. The anti-apoptotic effect is mainly mediated by competitive binding to caspase 8 (48).

The first step was to validate the results obtained in the transcriptional microarray time series on FOXO1A, IER3 and CFLAR. Using the same temporal conditions in an independent experiment, their expression levels were measured by RT-QPCR (Figure 2A). Microarrays and RT-QPCR time series exhibit similar time profiles and confirmed that EWS-FLI1 down-regulates these genes. Based on the literature mining used for the influence network reconstruction (fact sheet, Supplementary Tables S7 and S8), their possible regulators were identified (Figure 6A). FOXO1A is regulated by E2F1 (49); IER3 is regulated by MYC, EP300, NFKB (RELA, NFKB1) (50) and CFLAR by NFKB (RELA, NFKB1) (51), and MYC (52). E2F2 and E2F5 were also investigated, as they are both modulated by EWS-FLI1 and share similarities with E2F1 (53).

The second step was to validate the results obtained in the transcriptional microarray time series on these regulators. Microarrays and RT-QPCR time series exhibited similar time profiles (Figure 2A and Supplementary Figure S6).

In the third step, regulators were individually and transiently silenced in shA673-1C inducible cell line. Expression levels of FOXO1, IER3, CFLAR and all regulators were measured by RT-QPCR after each silencing experiment (Supplementary Table S10).

All these RT-QPCR data were semi-automatically analyzed by a reverse engineering method as following (see ‘*Network reverse engineering from siRNA silencing data*’ in Materials and Methods):

- Identification of influences from experimental data (represented by all arrows of Figure 6B). Links from EWS-FLI1 are based on RT-QPCR time series; other links are extracted from siRNA/RT-QPCR experiments.
- Confrontation with the literature. Five out of seven influences were confirmed. The two remaining influences (E2F1 -| FOXO1 and P300 -| IER3) display opposite effects as the one described by the literature (Figure 6C) and were therefore modified in the final version of the influence network.
- Extraction of the necessary connections *using the influence subnetwork of point* (i), represented by solid arrows in Figure 6B. It is to notice that some influences cannot be interpreted. For instance IER3 can be either directly activated by RELA or indirectly activated through a double inhibition via P300 (RELA -| P300 -| IER3), see Figure 6D.
- Filtering the necessary connections identified in (iii) *using the complete network model in* Figure 4A. It consists of confronting all necessary connections of Figure 6B with the literature mining producing the influence network, as described in Table 4. Validity of this subnetwork is therefore confirmed with the exception of one unexplainable necessary connection (P300 -| E2F2). In case of conflict between an experimental observation and an interaction described in the literature, we always used the connection inferred from Ewing’s specific experimental data, because the original goal of this work is to construct the network model specific to the molecular context of Ewing’s sarcoma.

The final refined model (Figure 4B) is obtained by adding all necessary connections (from transcriptome time series and siRNA/RT-QPCR experiments) to our literature-based network. Altogether, our results demonstrate the coherence of this influence network model describing EWS-FLI1 impact on cell cycle and apoptosis. Importantly, successive steps allowed to identify novel players involved in Ewing sarcoma such as CUL1 or CFLAR or IER3.

**Figure 5.**
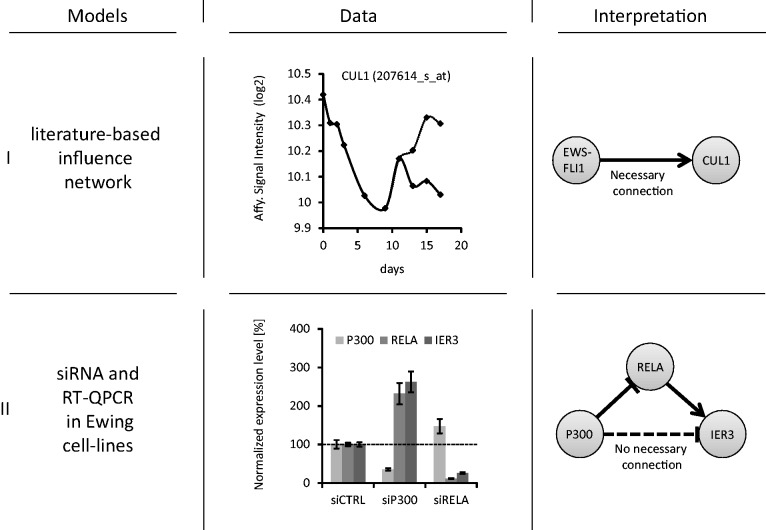
Illustration of necessary and non-necessary connections within given network models and data. (i) An observed influence from EWS-FLI1 to CUL1 is a necessary connection because no indirect explanation (path with intermediate nodes) can be identified within the network model. (ii) P300 represses IER3, but this can be explained through RELA, thus P300 -| IER3 is not necessary.

**Table 4.** siRNA/RT-QPCR data confronted to the network; each necessary connection from the network shown in Figure 5B (plain arrows) is confronted to the global EWS-FLI1 signaling network (Figure 3A)

Type	Connection	Possible intermediate node	Comment, possible scenario
	EWS-FLI1 → E2F1	E2F2, with E2F2 → E2F1	Possible scenario through cyclin and RB
	EWS-FLI1 → E2F2	P300, with p300 -| E2F2	EWS-FLI1 -| IER3 -| P300
			Necessary connection identified by transcriptome time series appears to be non-necessary
	EWS-FLI1 -| CFLAR	MYC, with MYC -| CFLAR	EWS-FLI1 → MYC
	EWS-FLI1 → E2F5	E2F2, with E2F2 → E2F5	
	E2F2 -| EP300	IER3, with IER3 -| EP300	E2F2 →(RBL.) -| MYC -| IER3
	IER3 -| EP300	RELA, with RELA -| EP300	IER3 → MAPK → TNF → NFKB
Necessary	EP300 -| E2F2	No other known transcriptional regulation (except EWS-FLI1)	
	P300 -| CREBBP	MYC, with MYC -| CREBBP	P300 -| E2F2 → RBL1 -| MYC
	IER3 -| CREBBP	MYC, with MYC -| CREBBP	IER3 → MAPK → MYC
	MYC -| CREBBP	P300, with p300 -| CREBBP	MYC → CCND → (E2F4,5:RBL2^P) → E2F4,5 → P300
	E2F1 -| MYC	E2F5, with E2F5 -| MYC	Cell cycle machinery: E2F1 → Cycle E → (E2F4,5:RBL2^P) → E2F4,5
	P300 -| MYC	E2F5, with E2F5 -| MYC	P300 →E2F2→E2F5
			Post-transcriptional effect of p300 on E2F2 may be stronger than transcriptional inhibition
	E2F5 -| MYC	P300, with p300 -| MYC	E2F5 → E2F5^p → P300
	MYC -| E2F1	E2F4, with E2F4 -| E2F1	MYC → CCND → (CCND:CDK)→ (E2F4,5:RB^p) → E2F4,5
	P300 -| E2F1	E2F4, with E2F4 -| E2F1	P300 → E2F4
	E2F1 -| NFKB1	P300, with P300 -| NFKB1	E2F1 → CCND3 → (CCND3:CDK) → (E2F4,5:RBL) → E2F4,5 → P300
	NFKB1 → E2F5	E2F2, with E2F2 → E2F5	NFKB → CCND1,2 → CCND:CDK → E2F1,2,3:RB^p → E2F1,2,3
	CREBBP → FOXO1	E2F1, with E2F1 → CREBBP	CREBBP → (E2F.)
	P300 -| RELA	E2F5, with E2F5 -| RELA	P300 →E2F2→E2F5
			Post-transcriptional effect of p300 on E2F2 may be stronger than transcriptional inhibition
	MYC -| RELA	E2F5, with E2F5 -| RELA	MYC → CCNE (or CCND) → CCNE:CDK → E2F4,5:RBL^p → E2F4,5
	E2F5 -| RELA	P300, with p300 -| RELA	E2F4,5 → p300
	RELA -| CFLAR		Published

For each of these connections, possible transcriptional regulators are identified from the ‘fact sheet’. For each possible transcriptional regulator, the shortest path between the source node of the connection and the regulator has been searched. If the sign of influence of the found path is compatible with the necessary connection, the path is considered as a ‘possible scenario’. Connections with mention ‘necessary’ in first column are considered as necessary related to siRNA/RT-QPCR data and to EWS-FLI1 network (Figure 3A), i.e. no coherent possible scenario has been found.

## DISCUSSION

We present in this article a molecular network dedicated to molecular mechanisms of apoptosis and cell cycle regulation implicated in Ewing’s sarcoma. More specifically, transcriptome time-series of EWS-FLI1 silencing were used to identify core nodes of this network that was subsequently connected using literature knowledge and refined by experiments on Ewing cell lines. For the construction of the network, no ‘a priori’ assumptions regarding the activity of pathways were made. In this study, EWS-FLI1-modulated genes are identified because they vary consistently along the entire time-series although they may have moderate amplitude. In comparison, the standard fold change-based approach focuses on the genes showing large variability in expression. For instance, CUL1 would not have been selected based on its fold change value (Figure 3B). The influence network is provided as a factsheet that can be visualized and manipulated in Cytoscape environment (37,54) via BiNoM plugin (28). The advantage of this approach is its flexibility. Indeed, the present model is not exhaustive, but rather a coherent basis that can be constantly and easily refined. We are aware that many connections in this model can be indirect. The network is a rough approximation of the hypothetically existing comprehensive network of direct interactions. More generally, we think that our method for data integration and network representation can be used for other diseases, as long as the causal genetic event(s) has(ve) been clearly identified.

### Biological implications

To validate the proposed network model, a dozen of EWS-FLI1 modulated transcripts and proteins were validated in shA673-1C cells as well as in four other Ewing cell lines. These additional experiments emphasized the robustness of our network to describe EWS-FLI1 effect on cell cycle and apoptosis in the context of Ewing sarcoma. Furthermore, the concept of *necessary connection* allowed to use this network for interpreting our experiments and identifying new connections. Our approach is therefore a way to include yet poorly described effects of EWS-FLI1 (which influences 20 network nodes).

After further experimental investigation, EWS-FLI1 induction of CUL1 appeared to be direct. In addition, the necessary connection EWS-FLI1 induces PRKCB and EWS-FLI1 represses CASP3 have been recently reported as direct regulations (16,39). CASP3 is shown here to be repressed by EWS-FLI1 in Ewing sarcoma cells. At the contrary, CASP3 is shown to be induced by ectopic expression of EWS-FLI1 in primary murine fibroblast (MEF) (16). This highlights the critical influence of the cell background on EWS-FLI1 mechanisms of action. MEF may not be the appropriate background to investigate in depth EWS-FLI1 properties. The notion of necessary connection enables to infer potential direct regulatory links between two proteins taking into account high-throughput data and a model of gene regulation extracted from the current literature. Considering EWS-FLI1 targets, it can therefore help designing specific experiments (ChIP or luciferase reporter experiments) to confirm or infirm direct regulations.

According to the ENCODE histone methylation profiles of several cell lines (55), the EWS-FLI1-bound CUL1 region appears highly H3K4me1 positive but H3K4me3 negative (Supplementary Figure 5B). H3K4 monomethylation is enriched at enhancers and is generally low at transcription start sites. By contrast, H3K4 trimethylation is largely absent from enhancers and appears to predominate at active promoters. This fits with our data indicating that EWS-FLI1 is direct enhancer of CUL1 and may be of particular interest in the context of cancer. Indeed, CUL1 plays the role of rigid scaffolding protein allowing the docking of F-box protein E3 ubiquitin ligases, such as SKP2 or BTRC, in the SKP1-CUL1-F-box protein (SCF) complex. For instance, it was recently reported that overexpression of CUL1 is associated with poor prognosis of patients with gastric cancer (56). Another example can be found in melanoma, where increased expression of CUL1 promotes cell proliferation through regulating p27 expression (57). F-box proteins are the substrate-specificity subunits and are probably the best characterized part of the SCF complexes. For instance, in the context of Ewing sarcoma, it was previously demonstrated that EWS-FLI1 promotes the proteolysis of p27 protein via a Skp2-mediated mechanism (58). We confirmed here in our time series experiment that SKP2 is down-regulated on EWS-FLI1 inhibition. Although SKP1-CUL1-SKP2 complex are implicated in cell cycle regulation through the degradation of p21, p27 and Cyclin E, other F-box proteins (BTRC, FBWO7, FBXO7 …) associated to CUL1 are also major regulators of proliferation and apoptosis [reviewed in (59)]. For instance, SKP1-CUL1-FBXW7 ubiquitinates Cyclin E and AURKA whereas SKP1-CUL1-FBXO7 targets the apoptosis inhibitor BIRC2 (60). SKP1-CUL1-BTRC regulates CDC25A (a G1-S phase inducer), CDC25B and WEE1 (M-phase inducers). Interestingly, the cullin-RING ubiquitin ligase inhibitor MLN4924 was shown to trigger G2 arrest at subsaturating doses in several Ewing sarcoma cell lines. This arrest could only be rescued by WEE1 kinase inhibition or depletion (61). In addition, *in vivo* preclinical data emphasized the potential antitumoral activity of MLN4924. Therefore, EWS-FLI1 regulation of CUL1 expression may profoundly affect SCF-mediated protein degradation and participate to proliferation and apoptosis deregulation in Ewing sarcoma.

An additional key player of oncogenesis is MYC. According to our results, MYC transcript was down-regulated by siRNA against EWS-FLI1 in all tested cell lines (including shA673-1C: supplementary Table S10 and Figure 2A). However, milder EWS-FLI1 silencing (DOX-treated shA673-1C cells) had more subtle influence on MYC transcript (Figure 2A) though the protein level was clearly decreased (Figure 2B). A post-transcriptional regulation may therefore be involved in the regulation of MYC by EWS-FLI1. In that respect, it is noteworthy that mir145, which represses MYC (62), was significantly up-regulated in DOX-treated shA673-1C cells (63) and could hence mediate this regulation. This justifies improving our network in the future including miRNA data.

**Figure 6.**
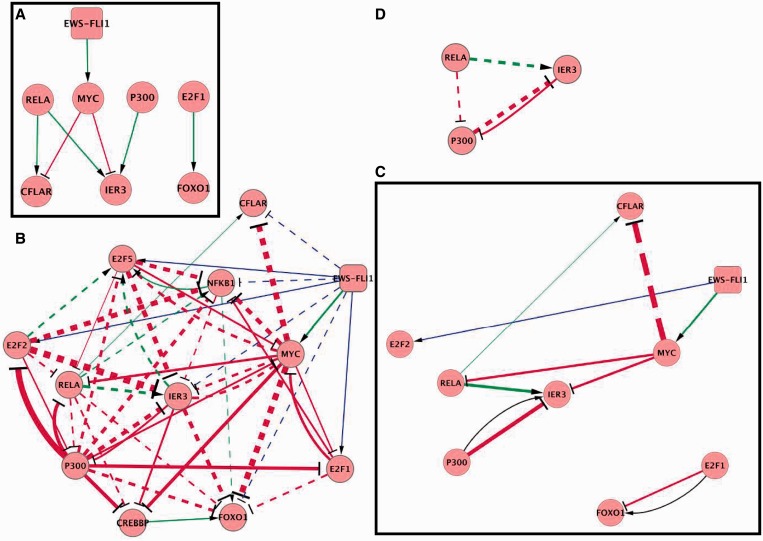
(**A**) Transcriptional influences between EWS-FLI1, CFLAR, MYC, P300, E2F1, RELA, IER3 and FOXO1 nodes extracted from the literature-based influence network. (**B**) Interpretation of experiments (siRNA transfection and RT-QPCR) in shA673-1C cells. Thickness of arrows shows the strength of the influence (values given in Supplementary Table S10). Blue arrows are based on RT-QPCR time series. Plain arrows represent transcriptional influences that are necessary for explaining data. Dashed arrows are questionable influences that can be explained through intermediate node. The arrow EWS-FLI1 -| FOXO1 is not necessary, although a recent article has identified it as a direct connection (72). (**C**) The necessary connections shown in Figure 6B have been compared with a subpart of the influence network (Figure 6A). All connections of this subpart have been confirmed, although two of them display an opposite sign. (**D**) Example of influences that cannot be interpreted as a necessary connection, because of ambiguity in the choice. Indeed, either RELA → IER3 is necessary and RELA -| P300 is not, or RELA-|P300 is necessary and RELA → IER3 is not. In this case, we decided to consider both connections (RELA → IER3; RELA -| P300) as non-necessary. Within this choice, the set of necessary connections is interpreted as the minimal set of connections that explain the maximum amount of data, with no ambiguity.

With the aim to experimentally validate a subpart of our influence network, regulators of IER3, CFLAR and FOXO1 were investigated. Importantly, most of the influences, taken from the literature, on these three genes were confirmed using siRNA/RT-QPCR experiments (Figure 6B and supplementary Table S10). The influences of P300 on IER3 and E2F1 on FOXO1 were found to be repressive (activating according to literature). Therefore, these influences were modified accordingly to our experimental data to fit to the context of Ewing sarcoma.

More interestingly, although P300 (in this study) and MYC (in this study and in the literature) repress IER3, IER3 most significant and yet unreported repressors are E2F2 and E2F5 (Figure 6B and Supplementary Table S10). This mechanism is enhanced through a synergistic mechanism of E2F2 on E2F5 (E2F2 -| IER3 and E2F2 → E2F5 -| IER3). Additionally, a positive feed-back loop is observed between IER3 and E2F5 (IER3 → E2F5) (Figure 6B and Supplementary Table S10). Therefore, it seems that these E2Fs play a major role in the regulation of IER3. Because IER3 is a modulator of apoptosis through TNFalpha or FAS-signaling (47), the balance between its repression (through MYC, E2F2 and E2F5 that are EWS-FLI1 induced and therefore disease specific) and activation (through NFkB) may be of particular interest in Ewing sarcoma. Indeed, suppressing NFkB signaling in Ewing cell line has been shown to strongly induce apoptosis on TNFalpha treatment (17).

All cell lines but EW7 carry p53 alterations. In patients, such mutations clearly define a subgroup of highly aggressive tumors with poor chemoresponse and overall survival (64,65). Most of the results obtained in EW7 cells were consistent with data from other tested cell lines except for its poor survival capacity on EWS-FLI1 knock-down (Supplementary Figure S4). However, procaspase 3 protein was not induced in EW7 cells on EWS-FLI1 knock-down (Figure 2B). Similarly, the two anti-apoptotic factors CFLAR and IER3 were only moderately up-regulated or even repressed after silencing of EWS-FLI1 in EW7 cells, respectively (Figure 2A). Since EW7 is one of the very few p53 wild-type celle line, these data may point out to some specific p53 functions in the context of Ewing cells.

### Perspectives

Owing to the flexibility of our network description format, further versions of the network will be produced. For instance, additional genomic data such as primary tumor profiling and ChIP-sequencing will be used to select new pathways for completing our network. Furthermore, regulated pathways such as Notch, Trail, hypoxia and oxidative stress regulation, Wnt or Shh identified in other studies could also be included (66–71). Finally, future experiments implying additional phenotypes (such as cell migration, cell–cell contact, angiogenesis …) could complete the present network.

It has to be noticed that our EWS-FLI1 network is not able to reproduce all the siRNA/RT-QPCR data: indeed some influences cannot be translated in terms of necessary connections, like in the example of Figure 6D. Therefore, this final network should be interpreted as the minimal one that reproduces the maximum amount of influences. We can suggest two methods for solving this problem of ambiguous interpretation: (i) extending experimental data by performing double-knockdown; (ii) comparing data to a mathematical model applied to the whole network, in a quantitative way. We can expect that new biological data and/or modeling results will help to enhance this network model, using the suggested framework of influence network and the concept of necessary connections. For instance, we believe that considering more complex patterns of expression response can be the next step in refining the Ewing’s sarcoma network. It may require increasing the number of experimentally measured time points.

Experimental results were confronted with literature knowledge within this network model. In particular, structural path analysis of the influence network was carried out to generate the Table 4; this can be considered as a simple theoretical approach. To obtain a predictive model, more sophisticated theoretical models will be constructed using the network, as already proposed in other systems biology approaches (73). However, this task can be complicated due to the size of networks: dynamical models often deal with <50 nodes to produce robust predictions. For such a network, there will be two types of strategies: (i) Considering only static network properties (steady states, through well-developed Flux Balance Analysis); (ii) Decompose the network into modules that will be modeled separately and then assembled into a modular network (74). More sophisticated modeling would help to overcome the two main limitations of the present approach, which are (i) EWS-FLI1-modulated genes have temporal expression profiles functionally similar to the dynamics of EWS-FLI1 expression, and (ii) interactions between genes and proteins are represented by influences (simple signed regulatory links).

The long-term goal is the construction of a theoretical model that fits heterogeneous experimental data (genomic, transcriptomic, proteomic in cell lines and primary tumors). In other words, we intend to construct a Ewing sarcoma-specific model, similarly to what has been done for liver cancer (75). Such a model should enable to propose (combination of) therapeutic strategie(s) specifically targeting phenotypes (such as proliferation and apoptosis induction).

## SUPPLEMENTARY DATA

Supplementary Data are available at NAR online.

## FUNDING

Institut National de la Santé et de la Recherche Médicale; Institut Curie; Agence National de la Recherche [SITCON project: NR-06-BYOS-0004]; Institut National du Cancer [SYBEwing project: 2009-1-PLBIO-04]; Ligue Nationale contre le Cancer (Equipe labellisée and CIT program); Réseau National des Génopoles; European Union (APOSYS, KCK and EET pipeline projects); société Française des Cancers de l’Enfant and the following associations: Courir pour Mathieu, Dans les pas du Géant, Olivier Chape, Les Bagouzamanon and les Amis de Claire. The research leading to these results has received funding from the European Union Seventh Framework Programme (FP7/2007-2013) ASSET project [FP7-HEALTH-2010-259348]. Funding for open access charge: Institut Curie.

*Conflict of interest statement*. None declared.

## Supplementary Material

Supplementary Data
