# Supplementary material for: Systems biology of Ewing sarcoma: a network model of EWS-FLI1 effect on proliferation and apoptosis
Source: Nucleic Acids Res. 2013 Aug 8;41(19):8853–71. doi: 10.1093/nar/gkt678 (PMC3799442; doi:10.1093/nar/gkt678)
Supplement: Supplementary Data [file supp_41_19_8853__index.html]

Systems biology of Ewing sarcoma: a network model of EWS-FLI1 effect on proliferation and apoptosis — Systems biology of Ewing sarcoma: a network model of EWS-FLI1 effect on proliferation and apoptosis — Supplementary Data 

# Systems biology of Ewing sarcoma: a network model of EWS-FLI1 effect on proliferation and apoptosis

## 

files

**Files in this Data Supplement:**

- Supplementary Data - pdf file
- Supplementary Data - xlsx file
