# Supplementary material for: Systems biology of Ewing sarcoma: a network model of EWS-FLI1 effect on proliferation and apoptosis
Source: Nucleic Acids Res. 2013 Aug 8;41(19):8853–71. doi: 10.1093/nar/gkt678 (PMC3799442; doi:10.1093/nar/gkt678)
Supplement: Supplementary Data [file supp_gkt678_nar-01667-h-2013-File012.pdf]

A

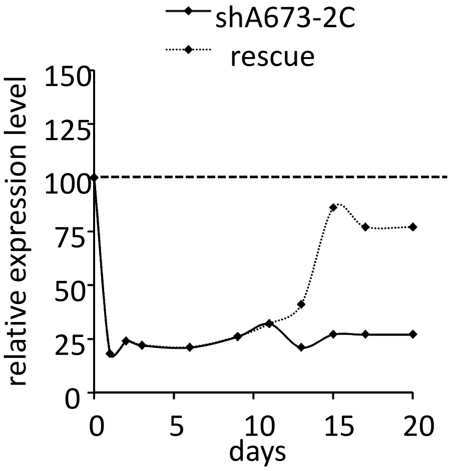

B

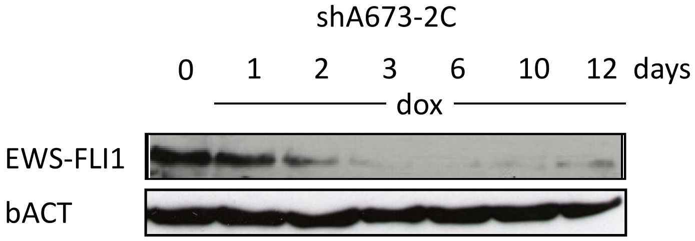

Supplementary Figure 1: (A) RT-QPCR of EWS-FLI1 in time series experiments in the shA673-2C inducible clone upon doxycycline addition/removal (plain: inhibition, dashed: rescue). (B) Western blot for EWS-FLI1 and beta-actin upon addition of doxycycline in the shA673-2C clone.

Histogram of fitting scores, for clone 1 and clone 2

Switch-like scores

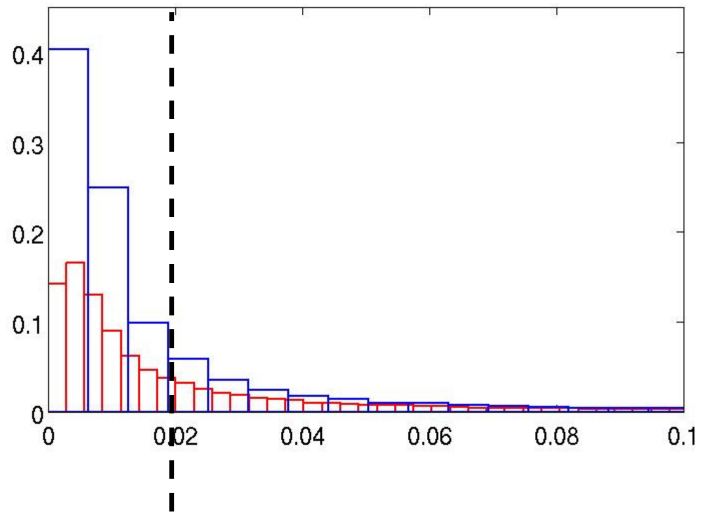

Threshold for selection=0.0242

Pulse-like scores

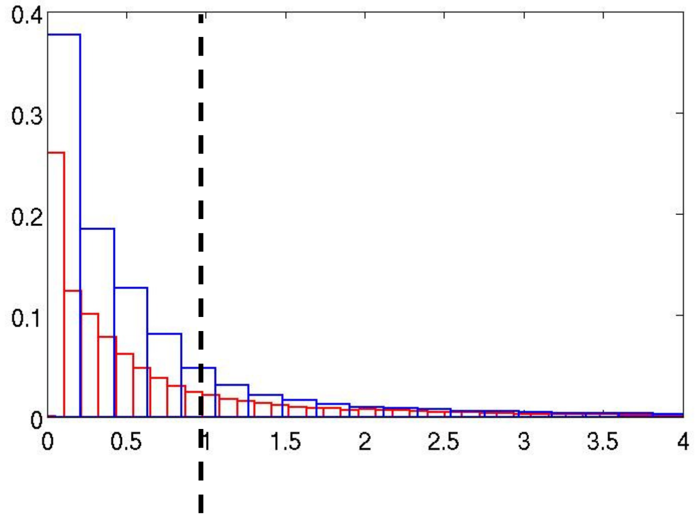

Threshold for selection=0.9389

Supplementary Figure 2. Histograms of fitting scores, for each non-linear fit (switch and pulse) and for each clone. Chosen thresholds are represented by a dashed line, they are based on visual inspection

Supplementary Figure 3: Concepts

Influence network: Network composed of positive and negative connections between nodes. The latter can represent mRNA, proteins or complexes. The connections can be transcriptional or post-transcriptional, direct or indirect, through several intermediate steps (unknown or absent in the network representation).

Necessary connection:

By definition, a necessary connection is such a regulatory connection between two molecular entities, which can be inferred from <u>the data</u> but cannot be predicted from <u>already existing network model</u>. From its definition, a necessary connection always depends on 1) dataset, and 2) already existing model. For instance, an observed influence between the node A and B becomes a necessary connection when no explanation (path with intermediate nodes) can be identified within the model (I). If an intermediate node C can explain this influence, then the connection becomes non-necessary (II). By increasing the data, for instance by silencing (using siRNA) the node C and measuring levels of A, B and C nodes (by RT-QPCR), two results are possible: the influence between A and B is retained, then A→B becomes a necessary connection (III); the influence between A and B is lost, then the connection is not necessary (IV).

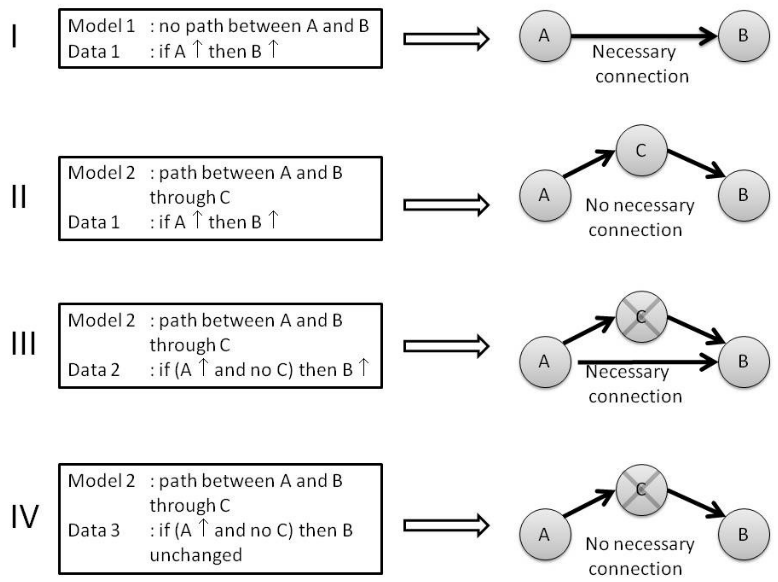

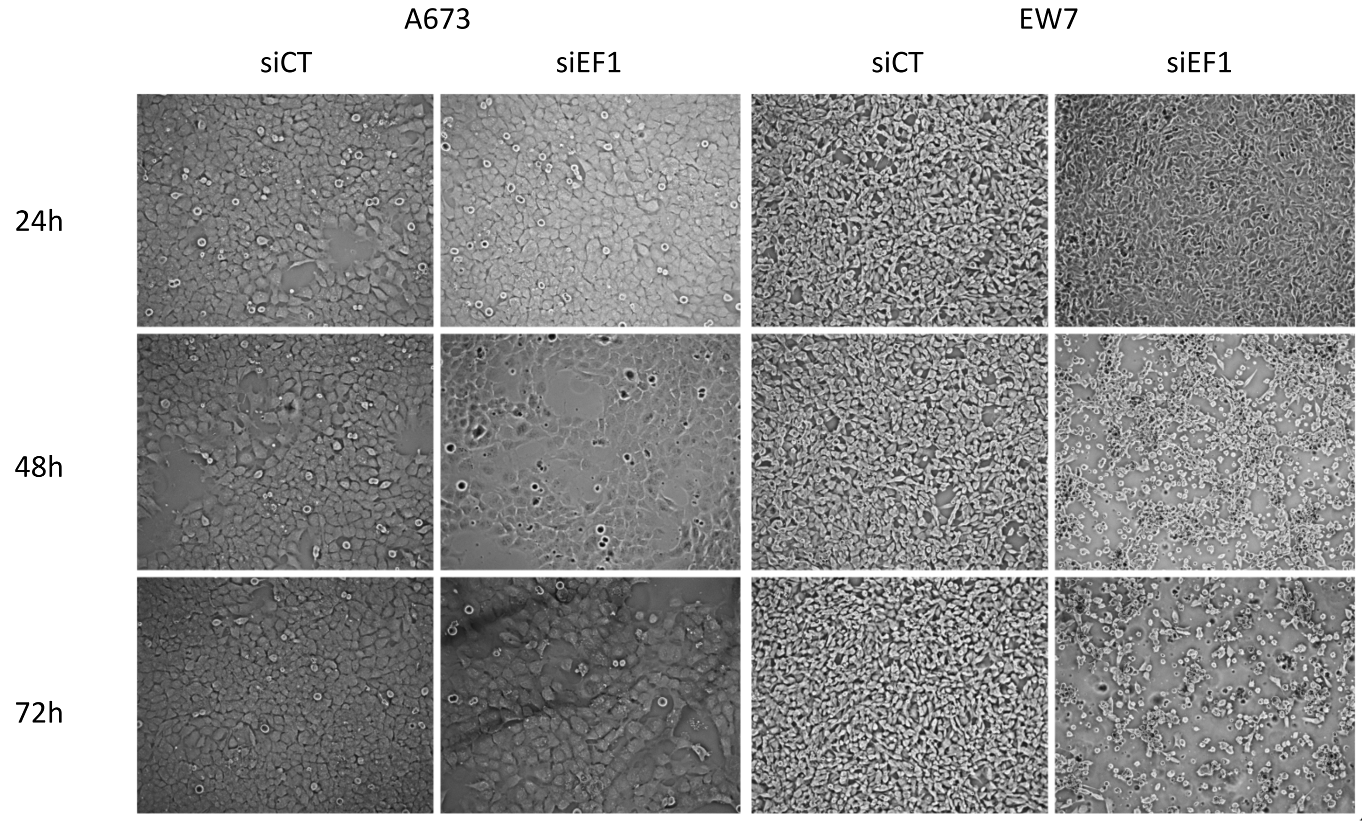

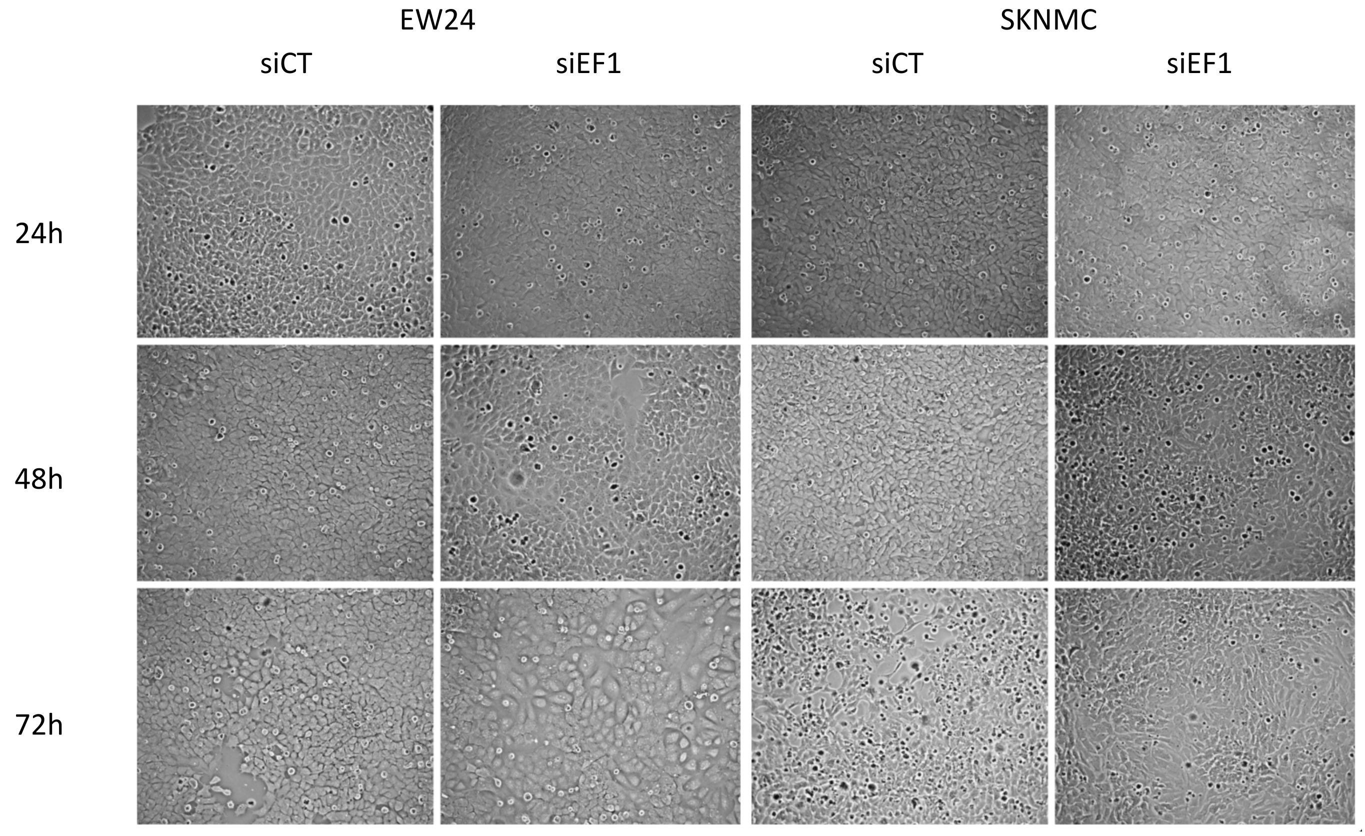

Supplementary Figure 4: Representative pictures of four Ewing cell lines upon transfection with siCT or siEF1 after 24, 48 or 72 hours.

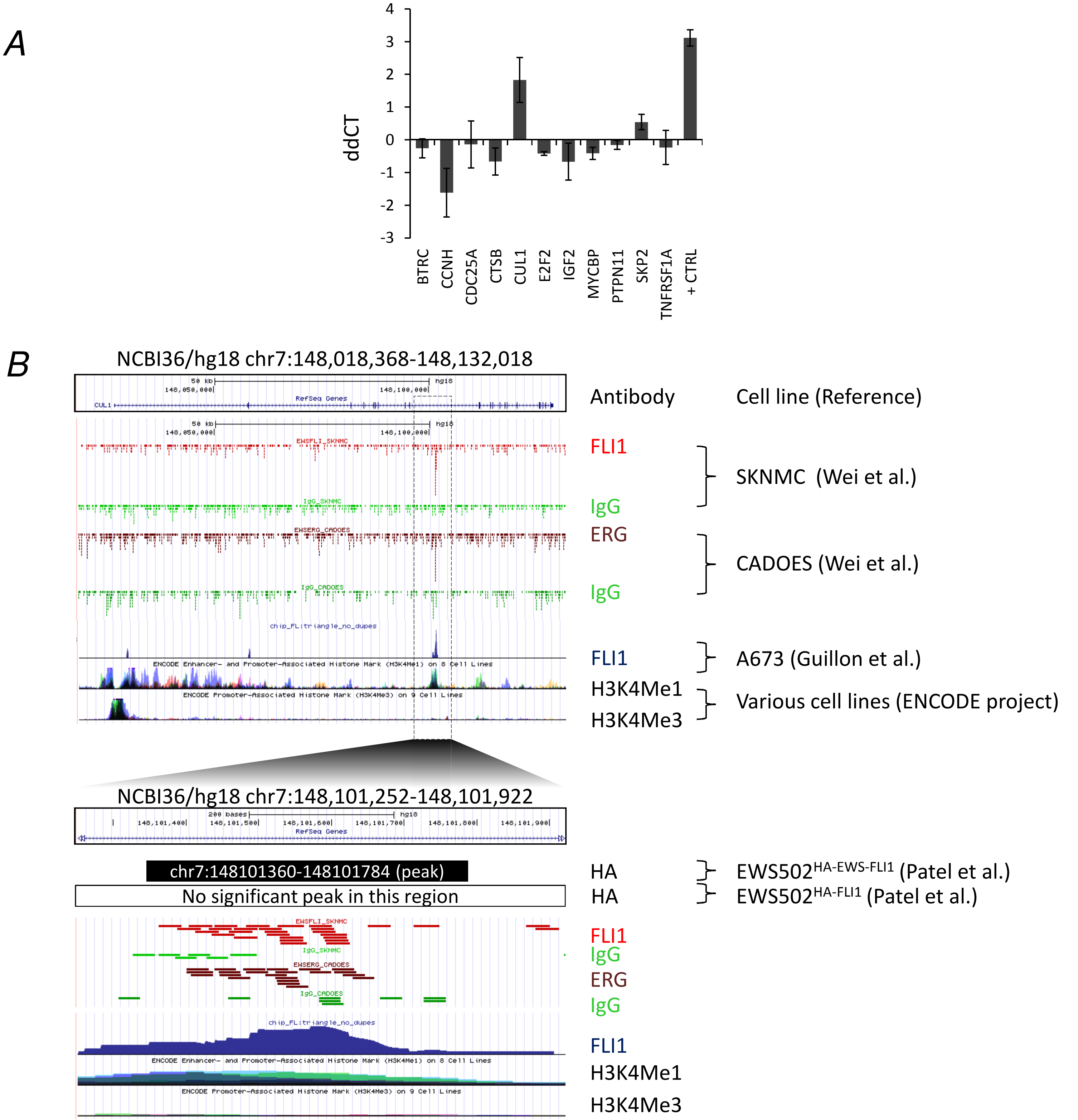

Supplementary Figure 5. (A) Necessary connection starting from EWS-FLI1 and displaying potential or low coverage ChIPSeq hits within the target genes were assessed by ChIP and QPCR experiments. Primers flanking these hits were designed and QPCR experiments were performed on either FLI1 or IgG antibody ChIP fractions. dCT was calculated between the ChIP and input CT. ddCT was calculated between FLI1 vs IgG conditions. CCND1 was used as CHIP positive control. Means and standard deviations of two experiments are shown. (B) Focus on the CUL1 gene: FLI1, ERG, IgG, H3K4me1 and H3K4me3 ChIPseq tags extracted from Wei et al., Guillon et al. and the ENCODE project were aligned to the human genome (NCBI36/hg18) and are displayed in the UCSC browser at two different magnification (top: whole CUL1 gene view; bottom: detail of the EWS-FLI1 binding region). This CUL1 ChIPseq peak is identified using three different antibodies: FLI1 antibody in A673 and SKNMC (EWS-FLI1 fusion type Ewing cells used in Guillon et al. and Wei et al.), ERG antibody in CADOES (EWS-ERG fusion type Ewing cell used in Wei et al.) and HA antibody in EWS502 (EWS-FLI1 fusion type Ewing cells in which EWS-FLI1 was persistently silenced and tagged HA-EWS-FLI1 (EWS502$^{HA\text{-}EWS\text{-}FLI1}$) or HA-FLI1 (EWS502$^{HA\text{-}EWS\text{-}FLI1}$) were reexpressed in Patel et al. (1)). In this last, a CUL1 CHIPseq peak is only identified in EWS502$^{HA\text{-}EWS\text{-}FLI1}$ cells but not in EWS502$^{HA\text{-}FLI1}$ cells.

1. Patel M, Simon JM, Iglesia MD, Wu SB, McFadden AW, Lieb JD, Davis IJ. (2012) Tumor-specific retargeting of an oncogenic transcription factor chimera results in dysregulation of chromatin and transcription. Genome Res, **22**(2):259-70.

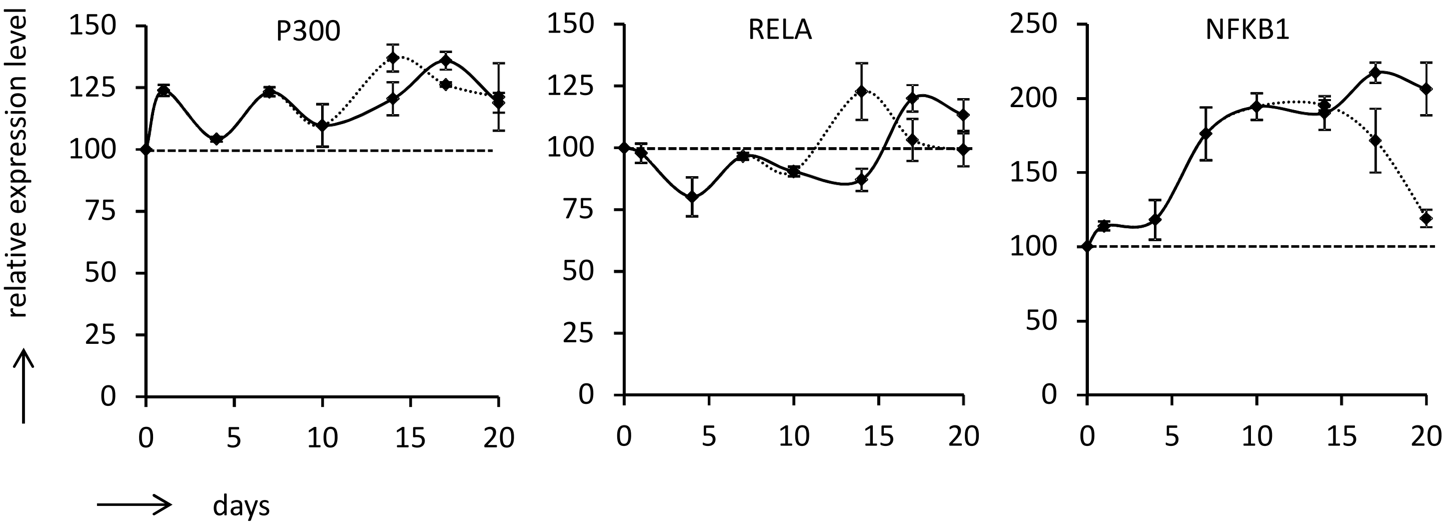

Supplementary Figure 6: RT-QPCR for P300, RELA and NFKB1 in time series experiments in the shA673-1C inducible clone upon doxycycline addition/removal (plain: inhibition, dashed: rescue) Data are presented as means with their standard error of the mean.

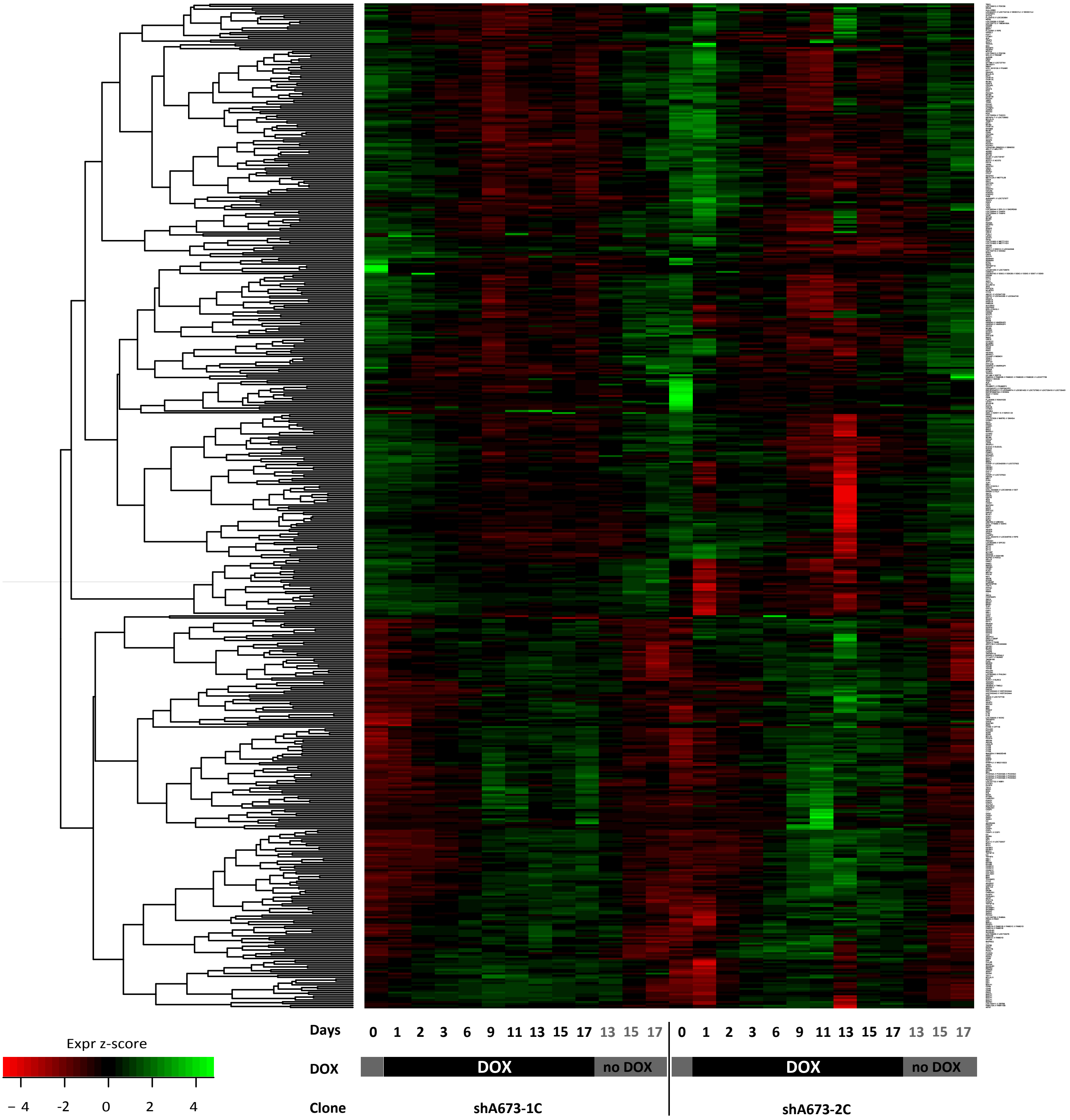

Supplementary Figure 7: Transcriptome heatmap representation of EWS-FLI1 modulated genes that related to apoptosis and/or proliferation.

Column names provide information about time points (in days) for EWS-FLI1 inihbition (by addition of DOX) and EWS-FLI1 inhibition-reactivation (DOX for 10 days followed by DOX removal) time-series experiments in shA673-1C and shA673-2C clones. Data are normalized by rows, therefore numerical values are z-scores.
